# Supplementary material for: Modeling glioblastoma heterogeneity as a dynamic network of cell states
Source: Mol Syst Biol. 2021 Sep 16;17(9):e10105. doi: 10.15252/msb.202010105 (PMC8444284; doi:10.15252/msb.202010105)
Supplement: Supplementary file 5 — Source Data for Figure 3 [file MSB-17-e10105-s001.zip › Figure3A_sourcedata/GSEA_3065/nosigdb_state3.GseaPreranked.1622718426028/GAROFANO_IAVARONE_NEU_SUBTYPE.html]

Details for gene set GAROFANO\_IAVARONE\_NEU\_SUBTYPE[GSEA]

|  || Dataset | state3 |
| Phenotype | NoPhenotypeAvailable |
| Upregulated in class | na\_pos |
| GeneSet | GAROFANO\_IAVARONE\_NEU\_SUBTYPE |
| Enrichment Score (ES) | 0.47057486 |
| Normalized Enrichment Score (NES) | 1.9553963 |
| Nominal p-value | 0.0 |
| FDR q-value | 8.18331E-4 |
| FWER p-Value | 0.006 |
Table: GSEA Results Summary

  

Fig 1: Enrichment plot: GAROFANO\_IAVARONE\_NEU\_SUBTYPE      
 Profile of the Running ES Score & Positions of GeneSet Members on the Rank Ordered List

  

| PROBE | GENE SYMBOL | GENE\_TITLE | RANK IN GENE LIST | RANK METRIC SCORE | RUNNING ES | CORE ENRICHMENT || 1 | BCAN |  |  | 0 | 0.883 | 0.0585 | Yes |
| 2 | GRIA2 |  |  | 38 | 0.460 | 0.0847 | Yes |
| 3 | RTN3 |  |  | 50 | 0.435 | 0.1122 | Yes |
| 4 | NCAM1 |  |  | 52 | 0.430 | 0.1406 | Yes |
| 5 | SOX6 |  |  | 88 | 0.358 | 0.1602 | Yes |
| 6 | CHD7 |  |  | 172 | 0.298 | 0.1703 | Yes |
| 7 | KIDINS220 |  |  | 187 | 0.291 | 0.1880 | Yes |
| 8 | NKTR |  |  | 202 | 0.282 | 0.2051 | Yes |
| 9 | ITM2C |  |  | 273 | 0.252 | 0.2136 | Yes |
| 10 | LSAMP |  |  | 307 | 0.241 | 0.2257 | Yes |
| 11 | ZEB2 |  |  | 401 | 0.218 | 0.2294 | Yes |
| 12 | FAM171A2 |  |  | 408 | 0.217 | 0.2430 | Yes |
| 13 | CADM2 |  |  | 416 | 0.215 | 0.2565 | Yes |
| 14 | NF1 |  |  | 425 | 0.214 | 0.2697 | Yes |
| 15 | WSB1 |  |  | 435 | 0.212 | 0.2827 | Yes |
| 16 | NAV2 |  |  | 486 | 0.201 | 0.2902 | Yes |
| 17 | REV3L |  |  | 506 | 0.198 | 0.3011 | Yes |
| 18 | DPP6 |  |  | 534 | 0.194 | 0.3108 | Yes |
| 19 | HIPK2 |  |  | 545 | 0.193 | 0.3224 | Yes |
| 20 | MTSS1 |  |  | 576 | 0.189 | 0.3315 | Yes |
| 21 | CASK |  |  | 643 | 0.180 | 0.3357 | Yes |
| 22 | GPC2 |  |  | 648 | 0.179 | 0.3471 | Yes |
| 23 | SLC35F1 |  |  | 711 | 0.168 | 0.3510 | Yes |
| 24 | SEC61A2 |  |  | 739 | 0.165 | 0.3588 | Yes |
| 25 | PAG1 |  |  | 761 | 0.162 | 0.3671 | Yes |
| 26 | DNM3 |  |  | 783 | 0.159 | 0.3752 | Yes |
| 27 | PODXL2 |  |  | 806 | 0.157 | 0.3831 | Yes |
| 28 | PHLPP1 |  |  | 819 | 0.156 | 0.3920 | Yes |
| 29 | RUFY3 |  |  | 848 | 0.153 | 0.3989 | Yes |
| 30 | GRIN2B |  |  | 890 | 0.149 | 0.4040 | Yes |
| 31 | PLXNB1 |  |  | 929 | 0.145 | 0.4092 | Yes |
| 32 | NIN |  |  | 933 | 0.145 | 0.4185 | Yes |
| 33 | PEG10 |  |  | 987 | 0.139 | 0.4215 | Yes |
| 34 | DAAM1 |  |  | 1211 | 0.121 | 0.4036 | Yes |
| 35 | ATCAY |  |  | 1327 | 0.113 | 0.3977 | Yes |
| 36 | CLN8 |  |  | 1340 | 0.112 | 0.4038 | Yes |
| 37 | CLASP2 |  |  | 1350 | 0.112 | 0.4101 | Yes |
| 38 | SRGAP3 |  |  | 1353 | 0.111 | 0.4173 | Yes |
| 39 | ARHGEF9 |  |  | 1402 | 0.108 | 0.4189 | Yes |
| 40 | ZNF300 |  |  | 1442 | 0.106 | 0.4214 | Yes |
| 41 | PCSK1N |  |  | 1484 | 0.104 | 0.4235 | Yes |
| 42 | YLPM1 |  |  | 1493 | 0.103 | 0.4294 | Yes |
| 43 | NLGN2 |  |  | 1506 | 0.103 | 0.4348 | Yes |
| 44 | KIF1A |  |  | 1583 | 0.099 | 0.4325 | Yes |
| 45 | ACVR2B |  |  | 1585 | 0.098 | 0.4389 | Yes |
| 46 | RERE |  |  | 1595 | 0.098 | 0.4444 | Yes |
| 47 | ATF7IP |  |  | 1612 | 0.097 | 0.4489 | Yes |
| 48 | USP24 |  |  | 1644 | 0.095 | 0.4516 | Yes |
| 49 | KIF3A |  |  | 1645 | 0.095 | 0.4579 | Yes |
| 50 | SPPL3 |  |  | 1681 | 0.093 | 0.4600 | Yes |
| 51 | ZNF462 |  |  | 1733 | 0.090 | 0.4600 | Yes |
| 52 | TSPAN7 |  |  | 1738 | 0.089 | 0.4655 | Yes |
| 53 | PTPRD |  |  | 1755 | 0.089 | 0.4695 | Yes |
| 54 | BMP7 |  |  | 1871 | 0.082 | 0.4616 | Yes |
| 55 | GABBR1 |  |  | 1892 | 0.081 | 0.4646 | Yes |
| 56 | KIF2A |  |  | 1899 | 0.081 | 0.4693 | Yes |
| 57 | SENP7 |  |  | 1962 | 0.078 | 0.4672 | Yes |
| 58 | POU2F1 |  |  | 1978 | 0.077 | 0.4706 | Yes |
| 59 | ARHGAP33 |  |  | 2065 | 0.073 | 0.4654 | No |
| 60 | MTMR9 |  |  | 2116 | 0.071 | 0.4643 | No |
| 61 | KIF5C |  |  | 2183 | 0.069 | 0.4613 | No |
| 62 | CSMD2 |  |  | 2213 | 0.068 | 0.4624 | No |
| 63 | MED13L |  |  | 2228 | 0.067 | 0.4652 | No |
| 64 | ABCC5 |  |  | 2250 | 0.066 | 0.4671 | No |
| 65 | DNER |  |  | 2341 | 0.063 | 0.4608 | No |
| 66 | NAV1 |  |  | 2451 | 0.059 | 0.4521 | No |
| 67 | SHC3 |  |  | 2460 | 0.058 | 0.4550 | No |
| 68 | ATG2B |  |  | 2469 | 0.058 | 0.4579 | No |
| 69 | KLHL24 |  |  | 2486 | 0.057 | 0.4598 | No |
| 70 | SPAST |  |  | 2560 | 0.055 | 0.4550 | No |
| 71 | NCOA2 |  |  | 2766 | 0.048 | 0.4344 | No |
| 72 | SGSM2 |  |  | 2780 | 0.048 | 0.4360 | No |
| 73 | ZMYM2 |  |  | 2782 | 0.048 | 0.4390 | No |
| 74 | FHIT |  |  | 2853 | 0.046 | 0.4339 | No |
| 75 | PPP1R12B |  |  | 2870 | 0.045 | 0.4351 | No |
| 76 | ZNF248 |  |  | 2876 | 0.045 | 0.4375 | No |
| 77 | RBPJ |  |  | 2897 | 0.044 | 0.4381 | No |
| 78 | MAPK8IP3 |  |  | 2937 | 0.043 | 0.4364 | No |
| 79 | LMBR1L |  |  | 2938 | 0.043 | 0.4392 | No |
| 80 | ABAT |  |  | 3059 | 0.040 | 0.4279 | No |
| 81 | PJA1 |  |  | 3102 | 0.038 | 0.4255 | No |
| 82 | FAM19A5 |  |  | 3130 | 0.038 | 0.4249 | No |
| 83 | FHOD3 |  |  | 3226 | 0.035 | 0.4162 | No |
| 84 | MAPT |  |  | 3333 | 0.032 | 0.4060 | No |
| 85 | RNF144A |  |  | 3368 | 0.031 | 0.4041 | No |
| 86 | C20orf194 |  |  | 3400 | 0.031 | 0.4025 | No |
| 87 | SSH2 |  |  | 3405 | 0.030 | 0.4041 | No |
| 88 | ZNF532 |  |  | 3576 | 0.026 | 0.3861 | No |
| 89 | GSK3B |  |  | 3606 | 0.025 | 0.3844 | No |
| 90 | PTBP2 |  |  | 3608 | 0.025 | 0.3859 | No |
| 91 | HIP1R |  |  | 3640 | 0.025 | 0.3840 | No |
| 92 | RASAL2 |  |  | 3679 | 0.023 | 0.3811 | No |
| 93 | CPEB4 |  |  | 3702 | 0.023 | 0.3800 | No |
| 94 | NXPH1 |  |  | 3773 | 0.021 | 0.3733 | No |
| 95 | TNIK |  |  | 3944 | 0.016 | 0.3546 | No |
| 96 | DCLK1 |  |  | 4019 | 0.015 | 0.3470 | No |
| 97 | DCLK2 |  |  | 4033 | 0.014 | 0.3464 | No |
| 98 | HMBOX1 |  |  | 4132 | 0.012 | 0.3358 | No |
| 99 | BRWD3 |  |  | 4139 | 0.011 | 0.3359 | No |
| 100 | ARVCF |  |  | 4211 | 0.009 | 0.3282 | No |
| 101 | FBXL16 |  |  | 4345 | 0.005 | 0.3131 | No |
| 102 | SBK1 |  |  | 4426 | 0.003 | 0.3040 | No |
| 103 | EZH1 |  |  | 4440 | 0.003 | 0.3027 | No |
| 104 | GRK4 |  |  | 4474 | 0.002 | 0.2990 | No |
| 105 | TSHZ1 |  |  | 4496 | 0.001 | 0.2966 | No |
| 106 | MAPRE2 |  |  | 4558 | -0.000 | 0.2896 | No |
| 107 | KIAA0895L |  |  | 4663 | -0.003 | 0.2777 | No |
| 108 | TSTD2 |  |  | 4667 | -0.003 | 0.2775 | No |
| 109 | VPS8 |  |  | 4692 | -0.004 | 0.2749 | No |
| 110 | FOXN3 |  |  | 4762 | -0.005 | 0.2672 | No |
| 111 | PRKAB2 |  |  | 4790 | -0.006 | 0.2645 | No |
| 112 | MMP15 |  |  | 4903 | -0.008 | 0.2520 | No |
| 113 | PHYHIPL |  |  | 4985 | -0.010 | 0.2433 | No |
| 114 | C2orf72 |  |  | 4986 | -0.010 | 0.2440 | No |
| 115 | GPT2 |  |  | 5114 | -0.014 | 0.2302 | No |
| 116 | EPHB2 |  |  | 5132 | -0.014 | 0.2291 | No |
| 117 | KIF26B |  |  | 5153 | -0.015 | 0.2278 | No |
| 118 | RALGPS2 |  |  | 5240 | -0.017 | 0.2189 | No |
| 119 | MIIP |  |  | 5262 | -0.017 | 0.2176 | No |
| 120 | TMOD2 |  |  | 5284 | -0.018 | 0.2163 | No |
| 121 | GADD45G |  |  | 5583 | -0.027 | 0.1834 | No |
| 122 | SATB1 |  |  | 5608 | -0.027 | 0.1825 | No |
| 123 | FAM107B |  |  | 5624 | -0.028 | 0.1826 | No |
| 124 | GSTA4 |  |  | 5649 | -0.028 | 0.1817 | No |
| 125 | EEPD1 |  |  | 5798 | -0.033 | 0.1666 | No |
| 126 | WWC1 |  |  | 5860 | -0.035 | 0.1619 | No |
| 127 | AKT3 |  |  | 5940 | -0.037 | 0.1551 | No |
| 128 | SMAP2 |  |  | 5966 | -0.038 | 0.1547 | No |
| 129 | WASF1 |  |  | 6025 | -0.040 | 0.1507 | No |
| 130 | CELF2 |  |  | 6152 | -0.045 | 0.1390 | No |
| 131 | CRMP1 |  |  | 6243 | -0.048 | 0.1317 | No |
| 132 | CDO1 |  |  | 6251 | -0.048 | 0.1341 | No |
| 133 | C5orf30 |  |  | 6285 | -0.049 | 0.1335 | No |
| 134 | ZMIZ1 |  |  | 6296 | -0.050 | 0.1357 | No |
| 135 | CLIP3 |  |  | 6655 | -0.064 | 0.0983 | No |
| 136 | CCDC28B |  |  | 6665 | -0.064 | 0.1015 | No |
| 137 | TMSB15A |  |  | 6711 | -0.067 | 0.1007 | No |
| 138 | NUDT11 |  |  | 6775 | -0.070 | 0.0979 | No |
| 139 | ZSWIM6 |  |  | 6802 | -0.071 | 0.0996 | No |
| 140 | RAB2A |  |  | 6844 | -0.073 | 0.0997 | No |
| 141 | GNG2 |  |  | 7035 | -0.083 | 0.0831 | No |
| 142 | RAP2A |  |  | 7092 | -0.086 | 0.0823 | No |
| 143 | UBL3 |  |  | 7332 | -0.103 | 0.0614 | No |
| 144 | EVL |  |  | 7641 | -0.132 | 0.0343 | No |
| 145 | RPAIN |  |  | 7805 | -0.153 | 0.0255 | No |
| 146 | CACNG4 |  |  | 8111 | -0.205 | 0.0036 | No |
| 147 | SOX4 |  |  | 8123 | -0.209 | 0.0162 | No |
| 148 | ENHO |  |  | 8124 | -0.210 | 0.0301 | No |
| 149 | MARCKSL1 |  |  | 8415 | -0.300 | 0.0163 | No |
| 150 | PFN2 |  |  | 8503 | -0.342 | 0.0288 | No |
Table: GSEA details [plain text format]

  

Fig 2: GAROFANO\_IAVARONE\_NEU\_SUBTYPE: Random ES distribution      
 Gene set null distribution of ES for **GAROFANO\_IAVARONE\_NEU\_SUBTYPE**
